# Effect of Multi-Pass Ultrasonic Surface Rolling on the Mechanical and Fatigue Properties of HIP Ti-6Al-4V Alloy

**DOI:** 10.3390/ma10020133

**Published:** 2017-02-06

**Authors:** Gang Li, Shengguan Qu, Mingxin Xie, Zhaojun Ren, Xiaoqiang Li

**Affiliations:** Department of Mechanical and Automotive Engineering, South China University of Technology, No. 381 Wushan Road, Tianhe District, Guangzhou 510640, China; ligangnuli@126.com (M.X.); renzhaojun71@163.com (Z.R.); Lixq@scut.edu.cn (X.L.)

**Keywords:** ultrasonic surface rolling, HIP Ti-6Al-4V, mechanical, fatigue, properties

## Abstract

The main purpose of this paper was to investigate the effect of a surface plastic deformation layer introduced by multi-pass ultrasonic surface rolling (MUSR) on the mechanical and fatigue properties of HIP Ti-6Al-4V alloys. Some microscopic analysis methods (SEM, TEM and XRD) were used to characterize the modified microstructure in the material surface layer. The results indicated that the material surface layer experienced a certain extent plastic deformation, accompanied by some dense dislocations and twin generation. Moreover, surface microhardness, residual stress and roughness values of samples treated by MUSR were also greatly improved compared with that of untreated samples. Surface microhardness and compressive residual stress were increased to 435 HV and −1173 MPa, respectively. The minimum surface roughness was reduced to 0.13 μm. The maximum depth of the surface hardening layer was about 55 μm. However, the practical influence depth was about 450 μm judging from the tensile and fatigue fracture surfaces. The ultimate tensile strength of the MUSR-treated sample increased to 990 MPa from the initial 963 MPa. The fatigue strength of the MUSR-treated sample was increased by about 25% on the base of 10^7^ cycles, and the lifetime was prolonged from two times to two orders of magnitude at the applied stress amplitudes of 650–560 MPa. The improved mechanical and fatigue properties of MUSR-treated samples should be attributed to the combined effects of the increased microhardness and compressive residual stress, low surface roughness, grain refinement and micro-pore healing in the material surface-modified layer.

## 1. Introduction

As a structural material, titanium alloys are widely applied in aerospace, automotive and biomedical engineering fields because of the unique combination of high strength-to-weight ratio, relatively low elastic modulus, good fatigue strength and toughness, and excellent corrosion resistance [1,2]. Titanium alloy components produced by Powder metallurgy (PM) technology can utilize the lower manufacturing costs compared to casting and forging methods. This will be helpful for enlarging the application fields of titanium alloy material. However, the products manufactured by the conventional powder sintering technology can not provide sufficient mechanical characteristics due to the residual porosity within the material. It has some obvious effects on tensile strength, Young’s modulus, strain-to-failure and fatigue-resistance characteristics of titanium alloy parts [3]. In addition, the residual pores will affect the cracks’ nucleation process due to the additional stress concentrators. As one of the powder sintering processes, the hot isostatic pressing (HIP) process uses high temperature and high pressure to manufacture parts. Therefore, it can avoid some material inner defects such as the uneven porosity and composition segregation introduced by other approaches [4,5], and the mechanical properties of the parts manufactured by HIP can be significantly improved [6,7]. Moreover, it also has some potential economic advantages of manufacturing complex shapes and fully dense components from titanium alloys using different wraps in the process of HIP [8]. In general, the fatigue failure of components will be accelerated by creating crack initiation sites on the surface. Moreover, the stress conditions on the machined surface are very important to material tensile properties [9]. In order to further improve material surface properties, some surface hardening methods (shot peening (SP) [10], surface mechanical attrition treatment (SMAT) [11], and deep rolling (DR) [12], or ultrasonic impact treatment (UIT) [13]) have been applied to produce severe plastic deformation in the material surface layer so as to change the microstructure, density and stress state of the material surface layer. Finally, the fatigue and tensile properties of the material produced by HIP will be discussed.

The main purpose of this work is to investigate the effect of multi-pass ultrasonic surface rolling (MUSR) on the fatigue and mechanical properties of Ti-6Al-4V alloy produced by HIP. MUSR treatment can effectively promote the microstructure evolution, grain subdivision and reorientation, and pore healing process accompanying material surface layer plastic deformation. Meanwhile, some micro-analytical instruments and techniques are used to characterize the microstructure and stress state change of the material surface modification layer, which will be responsible for the enhanced fatigue and mechanical properties of the MUSR-treated HIP Ti-6Al-4V alloy.

## 2. Experimental Details

### 2.1. Materials

**Figure 1:**
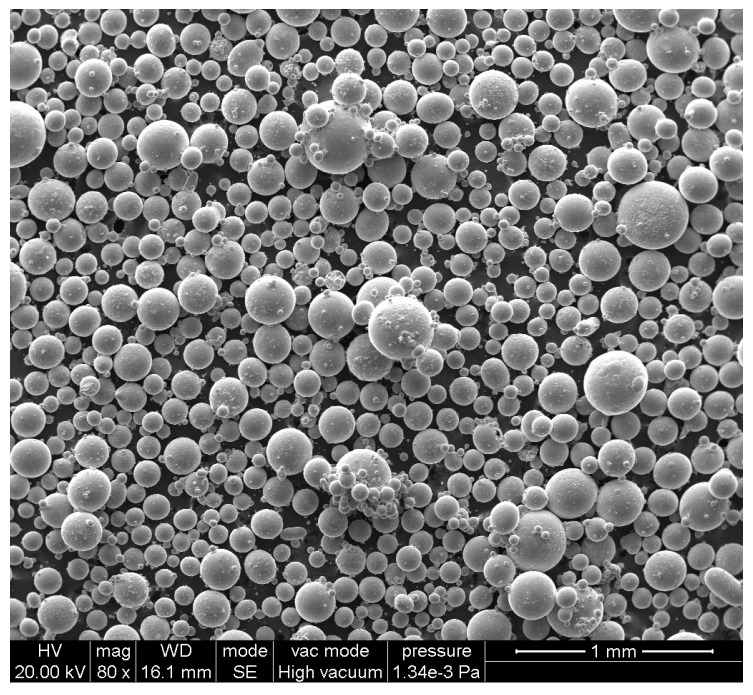
SEM image of the prealloyed powder manufactured by gas atomization.

The Ti-6Al-4V bar for MUSR treatment was manufactured by HIP, and the prealloyed powder used in this work was produced by the electrode induction melting gas atomization (EIGA) technique. The particle size of powder ranged from 50 to 400 μm and the average particle size was about 180 μm, as shown in Figure 1. The chemical composition of HIP Ti-6Al-4V alloy used in this experiment is shown in Table 1. The processing parameters of HIP were as follows: 850 °C (heating temperature), 50 MPa (vacuum pressure) and 3 h (time). The density of HIP bar was 99.8%.

### 2.2. MUSR Treatment

**Figure 2:**
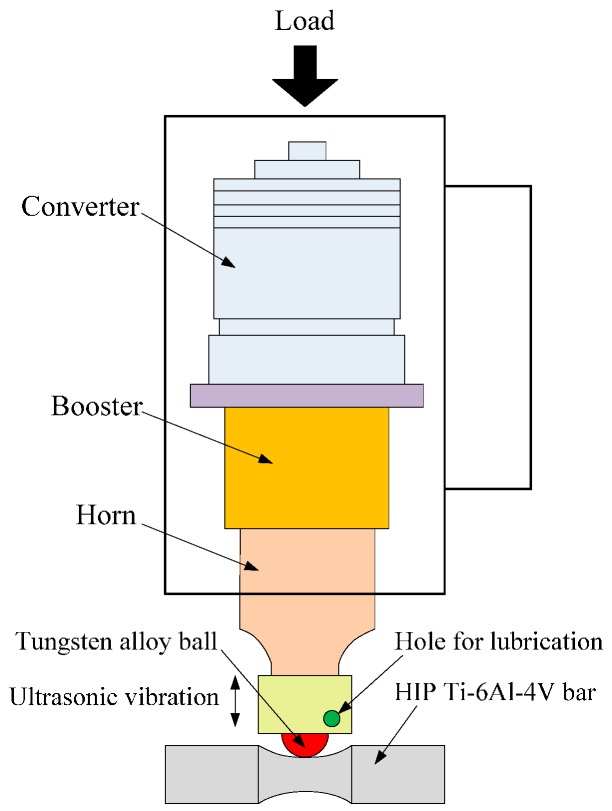
The schematic diagram of MUSR (multi-pass ultrasonic surface rolling) treatment.

MUSR treatment was conducted on a lathe using a carbide alloy rolling ball in the conditions of lubricating and pouring cooling fluid into the hole. The processing parameters of MUSR are shown in Table 2. The HIP Ti-6Al-4V bar was ultrasonic rolled six times using the same processing parameters. The rolling direction was unidirectional, and not reciprocating. The entire treatment was carried out at room temperature. The main principle of MUSR treatment is the use of resonant impulses generated by an ultrasonic generator into the harmonic shock of a work ball with a certain amplitude, as shown in Figure 2. MUSR treatment could provide the good surface quality and uniform surface-modified layer structure. The rolled ball could reduce the friction between ball and component and avoid the ploughing phenomenon generated by the fixed ball under the high load. Moreover, the non-fixed rolling tool facilitated replacement with other different shapes of rolling tools that were used for processing the different parts.

### 2.3. Tensile Tests

**Figure 3:**
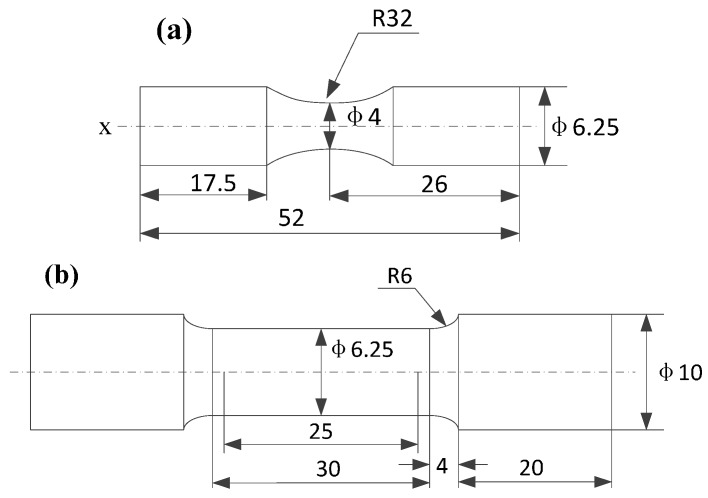
The schematic diagrams for tensile and fatigue samples: (**a**) fatigue sample; (**b**) tensile sample.

The tensile specimens were prepared according to ASTM (American Society for Testing and Materials): E-8 procedure, as shown in Figure 3b. Tensile specimens were cut from the HIP Ti-6Al-4V alloy bar, with a gauge length of 25 mm and a diameter of 6.25 mm. The tensile properties of the specimens before and after MUSR treatment were tested by an ultimate tensile testing machine (E45, SANS, Shenzhen, China) of 50 kN capacity at a speed of 20 mm/min at room temperature, and the stress and strain dates were obtained by an extensometer.

### 2.4. Fatigue Tests

The size of an hour-glass specimen for the fatigue test was shown in Figure 3a. It had a gauge length of 16 mm and a diameter of 4 mm in the net section. Stress controlled rotary bending fatigue tests for the specimens before and after MUSR treatment were conducted using a rotary bending machine (QBWP, Changchun, China) at a cyclic frequency of 100 Hz at ambient temperature. Stress amplitude $\sigma_{a}$ in the net section of the specimen was estimated by accounting for the balance of the inertia forces arisen at bending load [14]:

$$\sigma =\frac{M\cdot y}{I_{x}},$$

where σ is the bending stress; *M* is the moment about the neutral axis; *y* is the perpendicular distance to the neutral axis; *I_x_* is the second moment of area about the neutral axis *x*. The numbers of cycles (N_f_) to failure were measured at different stress amplitudes ($\sigma_{a}$) applied to the net section of the specimen. The fatigue life change trend of the studied specimens before and after ultrasonic surface rolling was presented as a Wöhler S–N plot, so the effect of ultrasonic surface rolling on material fatigue properties can be obviously seen. 

### 2.5. Microstructure Analysis

MUSR-treated samples for microscopic analysis were prepared using SiC abrasive papers progressively from 180 to 2500 grit. The samples then were polished the cross-section, etched in Kroll’s reagent, and finally washed ultrasonically in acetone. The cross-sectional microstructure was studied by SEM (Quanta 200 FEG, FEI, Hillsboro, OR, USA) with a voltage of 20 kV. TEM images and the corresponding selected area electron diffraction (SAED) were obtained by TEM (TECNAIG2S-TWINF20, FEI, Hillsboro, OR, USA) with an accelerating voltage of 200 kV. In order to measure the depth of surface hardened layer, the microhardness distribution along the depth direction from the treated surface was measured by Vickers microhardness tester (MVS 1000D1, Guangjing, Guangzhou, China) with a diamond Vickers indenter in the condition of a load of 0.25 N for a dwell time of 15 s. The residual stress values were measured by Rigaku Automate II equipment (Cu kα radiation, wave length of 1.54 ang, a tube current of 40 mA and a tube voltage of 30 kV, Tokyo, Japan) using the sin^2^ψ method, and the phase constituents were analyzed in the 2θ range 30°–80°.

## 3. Results and Discussion

### 3.1. Microstructure

**Figure 4:**
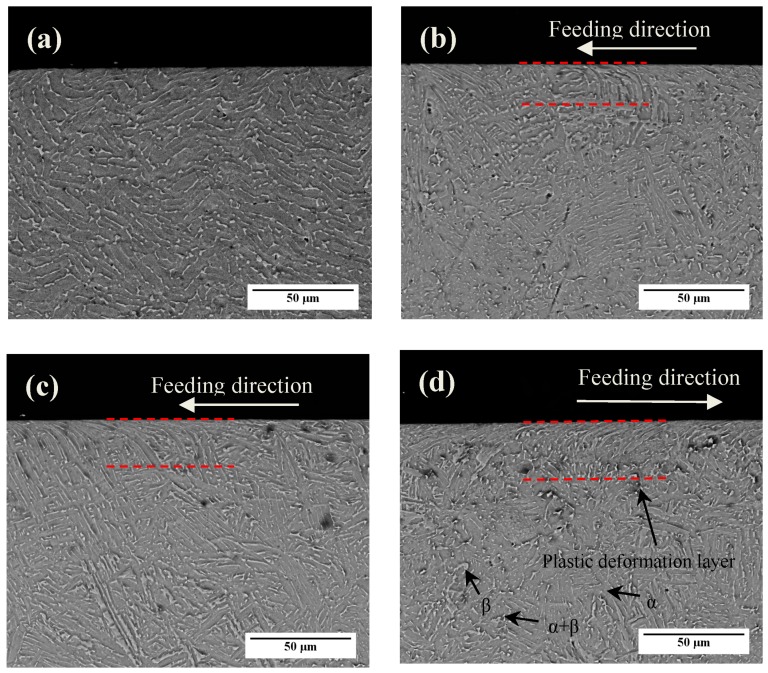
SEM photographs of the cross-section of HIP (hot isostatic pressing) Ti-6Al-4V samples before and after MUSR treatment: (**a**) untreated sample; (**b**) 30 kHz–600 N; (**c**) 30 kHz–750 N; (**d**) 30 kHz–900 N.

The cross-sectional microstructure images of HIP Ti-6Al-4V alloy before and after MUSR treatment are shown in Figure 4. MUSR-treated sample has an obvious plastic deformation in the top-surface layer compared to the untreated sample, and material deformation direction is consistent with the feed direction of rolling ball. The extent of material plastic deformation is gradually increased with the increase of load used by MUSR. There is no clear dividing line between the plastic deformation layer and inner matrix. MUSR treatment effectively promoted grain refinement and increased dislocation density in the material surface-modified layer [15]. The grains are elongated and become curved. There is a wave on the surface appearance after MUSR. MUSR treatment can change the part geometric allowances, but the effect of MUSR treatment on part geometry or dimension is very small and can be ignored. Most importantly, MUSR treatment can modify material surface layer microstructure, refine the composition phase and grain and produce plastic deformation along the feeding direction.

There are no obvious big holes in the relatively dense material plastic deformation layer. This should be attributed to the good combination effects of load and ultrasonic vibration. The maximum thickness of the material deformed layer observed by the eyes (between the red lines in Figure 4d) is about 35 μm. It is well known that material plastic deformation layers have a strong correlation with the microstructure and mechanical properties in many metallic materials [16].

**Figure 5:**
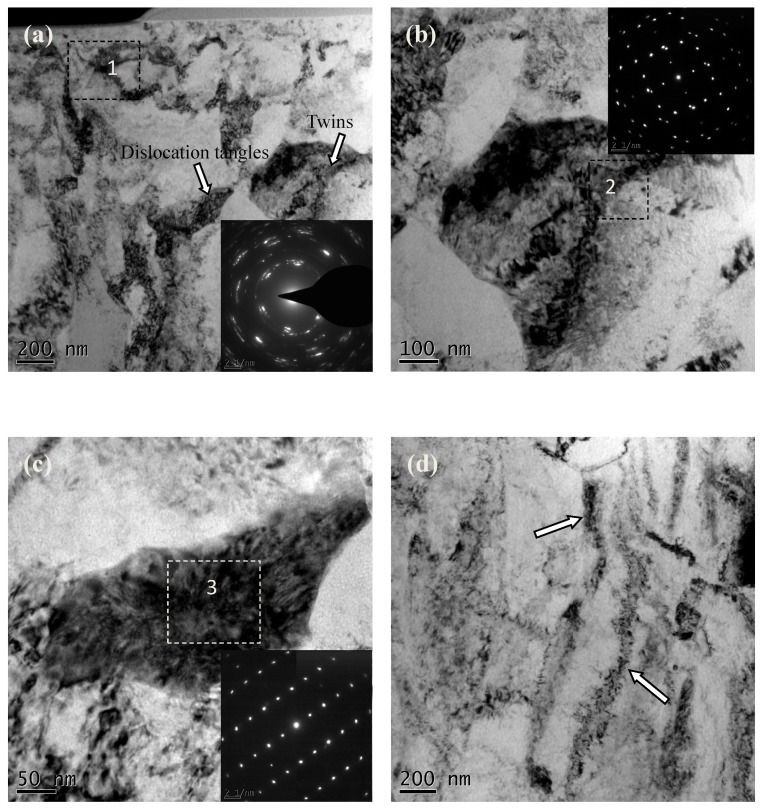
TEM images of sample treated by MUSR using 30 kHz and 900 N at the depth range of 2–6 μm from the treated surface: (**a**) the depth of 3 μm; (**b**) deformation twins; (**c**) dislocations tangles; (**d**) the depth of 6 μm.

TEM images corresponding to the surface-modified layer in Figure 4d are shown in Figure 5, and the depth range is 2 to 6 μm from the treated surface. As shown in Figure 5a, the grains in the top-surface layer are obviously refined and mostly identified as equiaxed with random orientations. The corresponding selected area electron diffraction pattern (SAED) (“1”) illustrates the coexistence of both grains and subgrains. Some dense dislocations can be found in the grain interior and boundaries that provide a high density of strain energy. The grain boundaries can be formed by the mode of annihilating, accumulating and rearranging of dislocations. Some boundaries are concurrently constituted by dislocation tangles and subboundaries. As strain and strain rate rise, the dislocation tangles will change into subboundaries. Thus, the refined grains will be formed in the intersections of subboundaries. Generally, dislocation tangles are formed in a complex way and mainly caused by tangling with each other. Material plastic deformation can be finished by dislocation motion at different depths from the treated surface. Once meeting some obstacles in some slip planes, dislocations will curve. Therefore, the dislocation densities there will increase accordingly and produce dislocation tangles [17]. The corresponding SAED (“3”) shows no misorientations inside the dislocation tangles in Figure 5c. The deformation twins can be identified by the SAED (“2”) of Figure 5b, which shows the symmetrical structure on both sides of twinning planes. Some dislocations also present in the interior of twins. The appearance of twins should be attributed to the relatively large plastic deformation and high strain rate produced by high-frequency MUSR. With the increasing of depth, the subgrains with low dislocation density turn into slip band structure (Figure 5d marked by white arrow).

**Figure 6:**
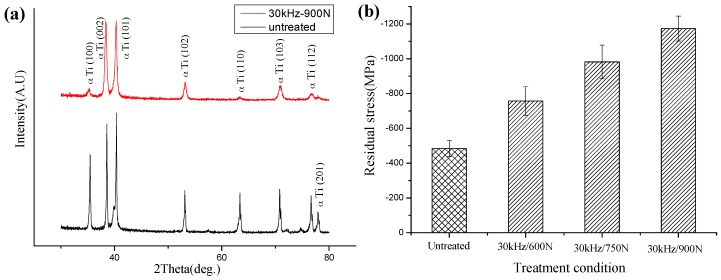
X-ray diffraction analysis: (**a**) X-ray spectra of the MUSR-treated sample at 900 N and the untreated sample; (**b**) compressive residual stress for the MUSR-treated sample at different loads and the untreated sample.

Prior to tensile and fatigue tests, X-ray diffraction measurements were conducted on the MUSR-treated and untreated samples to evaluate the magnitude of compressive residual stress, as well as the extent of work hardening induced in material surface layer by MUSR treatment. MUSR treatment significantly improved the levels of compressive residual stress in the material surface region nearby, and broadened the FWHM (full width at half maxima) of X-ray diffraction lines (Figure 6a). The maximum compressive residual stress introduced by MUSR in Figure 6b is as high as −1173 MPa. It is obviously higher than the residual stresses (−483 MPa) caused by cutting. FWHM is useful to characterize the degree of work hardening in mechanically surface treated materials [18]. Meanwhile, the high residual stress usually correlates well to material surface microhardness value. It is helpful to increase surface damage resistance of the material [19]. Essential growth of FWHM after MUSR treatment indicates the increase in dislocation density or lattice micro-strains and some diminution in grains size. It is well known that residual stress values gradually increase with the decrease of distance from the top surface. The increased compressive residual stress of MUSR-treated samples indicates that the plastic deformation in the material surface layer should be attributed to a large amount of dislocation generation. Therefore, it can be deduced that compressive residual stress extended to the depth corresponding to the dislocation generation region [20]. Compressive residual stress introduced by MUSR treatment in the material surface layer can reduce and negate any residual or subsequently imposed tensile stress at the material surface. Most fatigue failures and tensile fractures start at or near the surface stressed in tension. Therefore, by reducing the net tensile stress at or near the material surface, and crack initiation or formation can be delayed. Finally, it will be helpful to improve the fatigue life and tensile strength of the components [21].

### 3.2. Surface Roughness and Microhardness Profile

**Figure 7:**
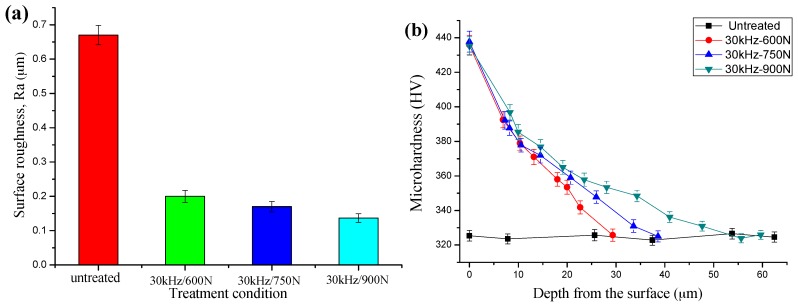
(**a**) Surface roughness values of the different conditions treated samples; (**b**) variation in microhardness of samples treated by MUSR along the depth direction from the treated surface.

Surface roughness change of the samples before and after MUSR treatment is shown in Figure 7a. Surface roughness of MUSR-treated samples is significantly lower than that of the untreated sample, and gradually decreases with the increase of load. The smallest value is 0.13 μm obtained by MUSR treatment at 900 N, which is almost one-sixth of the untreated sample. Such reduction in surface roughness is similar to that of the deep rolling process [10], but it is different from the shot peening process, which usually increases material surface roughness [22]. MUSR treatment can produce the shallower indents due to high transversal constituent load at the sliding impacts applied to the surface, as well as the high density impact points. The smooth surface caused by MUSR treatment can significantly improve the resistance of crack initiation in the material surface and change the state of stress concentration. Hence, this surface hardening technology can effectively increase the fatigue life and tensile strength of the samples under the same test conditions.

Microhardness profile of MUSR-treated and untreated samples along the direction of depth is shown in Figure 7b. It can be seen that the surface microhardness of the MUSR-treated sample is increased to 436 HV from the initial 325 HV. The increased surface microhardness should be attributed to the thick plastic deformation layer, good grain refinement and work-hardening effect in the material surface layer following the Hall–Petch relationship in addition to the large compressive residual stress introduced by MUSR in the material surface layer. These factors increase the material’s deformation resistance in the process of microhardness measurement. The high surface hardness can improve the situation of mechanical surface damage [23]. However, it can be seen that the increase of the load does not increase the micro-hardness at the surface. This can be explained by the small load used in the process of microhardness measurement. Therefore, it can not penetrate the material surface modification layer. The physical strengthening process to improve the surface hardness of the material is very limited at an ambient temperature due to the inherent material properties. When material surface microhardness reaches the maximum limit, the increased load used by MUSR can not change the change of surface microhardness. The depth of surface hardening layer is gradually increased with increasing of applying load used by MUSR. The maximum hardening layer depth is about 55 μm obtained by the load of 900 N. In the process of MUSR treatment, the rolling head will deliver the ultrasonic vibration energy on to the material surface modification layer. On the one hand, there occurs material obvious plastic deformation in the surface layer. On the other hand, material density also increases in the material surface layer. Therefore, the actual influence depth in the material surface is larger than the thickness of material plastic deformation. This also interprets why the depth of the material hardening layer is larger than the thickness of material obvious plastic deformation. The deep surface hardening layer and good grain refinement effects can change or delay surface micro-crack formation, and enhance material tensile and fatigue properties.

### 3.3. Tensile Behavior

**Figure 8:**
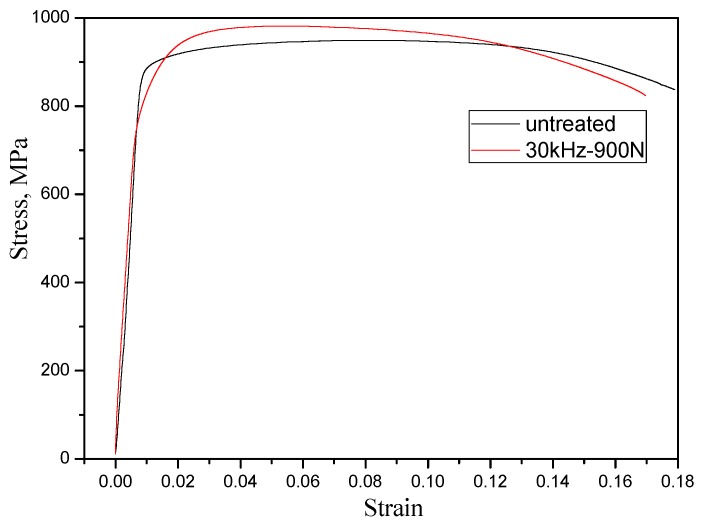
The stress strain curves for the MUSR-treated sample at 900 N and untreated sample.

The tensile tests were conducted to quantitatively measure the mechanical properties of HIP Ti-6Al-4V alloy before and after MUSR treatment. In general, the ultimate tensile strength, elongation and percentage reduction of area are considered as the main factors to evaluate the material ductility in static simple tension. The ultimate tensile strength of MUSR-treated sample at 900 N is increased to 990 MPa from the initial 963 MPa in Figure 8. Meanwhile, the elongation and reduction of area are 19% and 51.7%, respectively. In contrast, the elongation and area reduction obtained from the untreated sample are 22% and 41.7%, respectively. Therefore, judging from these dates, it can be deduced that the MUSR-treated sample has good tensile properties compared to the untreated sample. The main reasons are as follows: MUSR treatment creates a thin layer of high magnitude compressive residual stress at or near the material surface, which is balanced by a small tensile stress in the material’s deeper core. Meanwhile, it also produces the dense dislocations and increases the micro-strain in the material surface-modified layer. It is well known that the interaction of high density dislocations can improve the yield strength of material, and compressive residual stress can suppress the initiation and propagation of cracks along grain/phase boundaries and lessen the intergranular fracture [24]. According to the Hall–Petch equation, the yield strength of material is also related to the grain size. It is gradually increased with the decrease of grain size [25]. Therefore, the fine-grained modification layer of the MUSR-treated sample can improve the formation of micro-cracks at the surface and produce an instant tensile fracture from material internal initiation.

**Figure 9:**
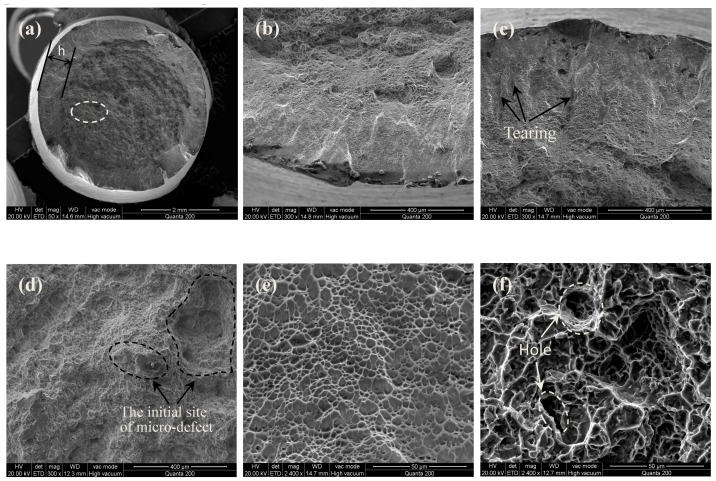
Tensile fracture morphologies of the MUSR-treated sample: (**a**) 50×; (**b**) cleavage; (**c**) tearing; (**d**) the crack initial site (white region in (**a**)); (**e**) dimples in the surface modification layer; (**f**) dimples and holes in the inner material substrate.

**Figure 10:**
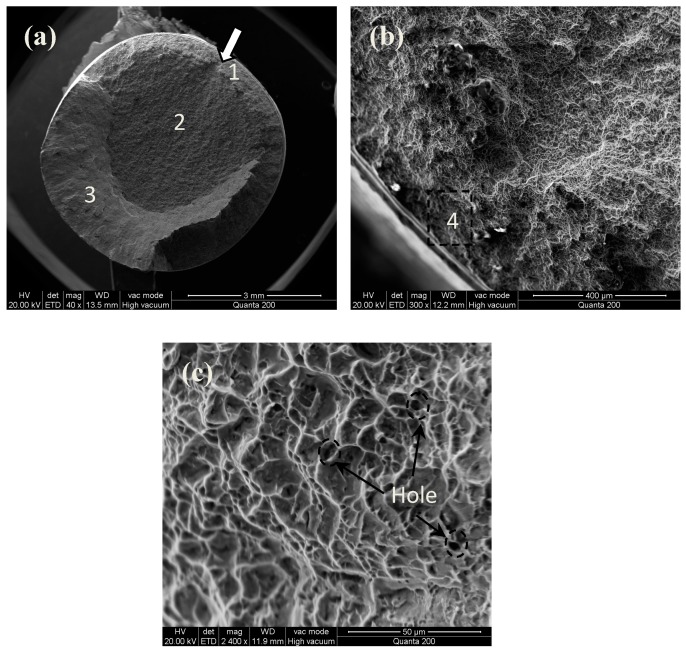
Tensile fracture morphologies of untreated specimen: (**a**) 40×; (**b**) the crack initial site; (**c**) the microstructure of area “4”.

Tensile fracture morphologies of the MUSR-treated sample at 900 N and the untreated sample are shown in Figure 9 and Figure 10, respectively. The MUSR-treated sample shows a different fracture morphology (Figure 9a) compared with that (Figure 10a) of the untreated sample. There is a transition layer (the thickness of h) from surface to inner substrate in Figure 9a. It can be seen as the actual affecting layer produced by MUSR treatment in the material surface layer. Although the obvious hardening layer is only tens of microns, ultrasonic impact and surface rolling can effectively heal material micro holes and reduce material micro defects below the surface hardening layer, thereby further increasing material density of the surface-modified layer—generally, the crack nucleates in the material surface, sub-surface, internal defects and stress concentration. However, the surface-modified layer obtained by MUSR treatment has refined grains, good strain strengthening and dense material, all of which can reduce the micro defects in the material surface layer. Therefore, compared to the material inner zone, the initial site of crack nucleation will not firstly appear in the material surface-modified layer. It may be located at the white region of Figure 9a (Figure 9d) due to material internal defects. However, the initial crack nucleation site of the untreated sample is near the material surface (indicated with the arrow in Figure 10a). The trace of crack development can be clearly seen in Figure 10b, and the final separation of the untreated sample is in an order “1”, “2” and “3”. It is obvious that there are some micro-pores in Figure 10c, which is the source of crack formation. In general, the tensile fracture mechanism is complicated rather than simple, and it can not be only decided by the single ductile or brittle fracture. As a typical characteristic of tensile fracture, cleavage steps can be seen in Figure 9b. Figure 9c shows the evidence of quasi-cleavage, which involves fine-scale mixtures of crystallographic cleavage and ductile tearing. There is a range of dimple appearance in Figure 9e,f, which can be considered as a type of ductile fracture. Figure 9f involves the more uniform and deeper cavities and some holes. There appears deep dimple aggregation, causing the improvement of the ductility. The existence of dimples in the fractograph means that crack propagation is accompanied by the local deformation process, but it cannot infer the ductile fracture in the macro range. The fractograph of Figure 9e is characterized with the fine equiaxed dimples generated by typical uniaxial tensiles. The dimples distributed in the surface-modified layer (Figure 9e) are shallower than those formed in the inner base material (Figure 9f), thus the tensile properties declined by a certain degree [26]. However, MUSR treatment can change the situation of the micro-crack initial nucleation in the material surface layer. These results mentioned above prove that the MUSR-treated sample has better tensile properties than the untreated sample.

### 3.4. Fatigue Behavior

**Figure 11:**
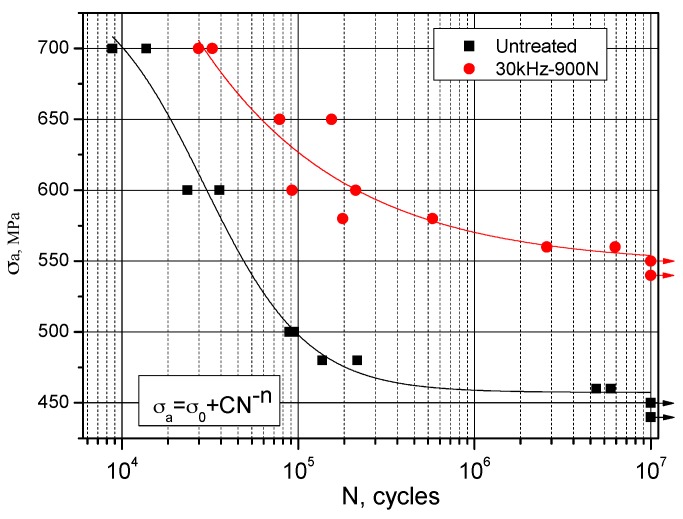
Wöhler curves for MUSR-treated samples at 900 N and untreated samples.

The stress/life fatigue behaviors of MUSR-treated samples at 900 N and untreated samples are compared in a form of Wöhler S–N curve in Figure 11. MUSR treatment leads to a significant increase in fatigue strength and enhances the fatigue lifetime, particularly in the high-cycle fatigue regime. Compared with the fatigue strength (465 MPa) of untreated samples, there is about a 25% increase for the fatigue strength (560 MPa) of MUSR-treated samples on the base of 10^7^ cycles. The main effect range of MUSR treatment on fatigue lifetime is between 560 MPa and 650 MPa. The increased degree of fatigue lives is gradually decreased with the increasing of applying stress, and changes from two orders of magnitude to two times. The beneficial influence of MUSR treatment on fatigue life prolongation in a high-cycle fatigue regime is very apparent. The Wöhler curves of MUSR-treated and untreated samples show the different slopes in low-cycle fatigue regions. The curve obtained by MUSR-treated samples becomes flatter, and this indicates higher resistance to overstressing. Moreover, the material after MUSR treatment becomes more resistant to microplastic deformation due to the surface layer strengthening effect compared to untreated material.

**Figure 12:**
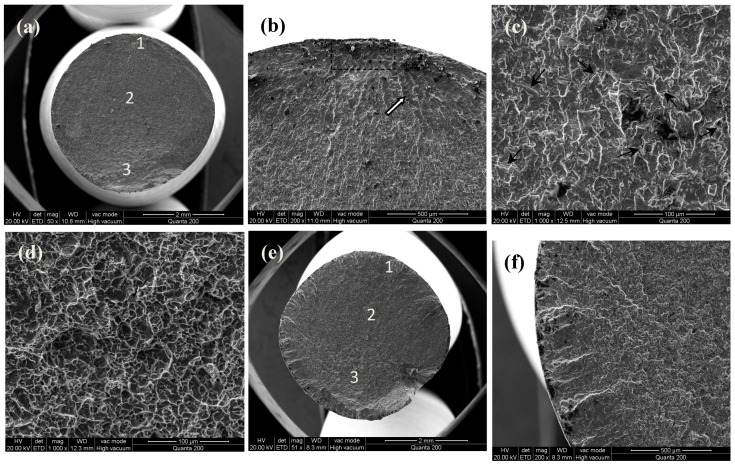
Fatigue fracture surfaces of untreated specimen at different stresses: (**a**) fracture surface at 450 MPa; (**b**) area “1” 200×; (**c**) local amplification of area “2”; (**d**) local amplification of area “3”; (**e**) fracture surface at 700 MPa; (**f**) cleavage striations in material surface layer.

**Figure 13:**
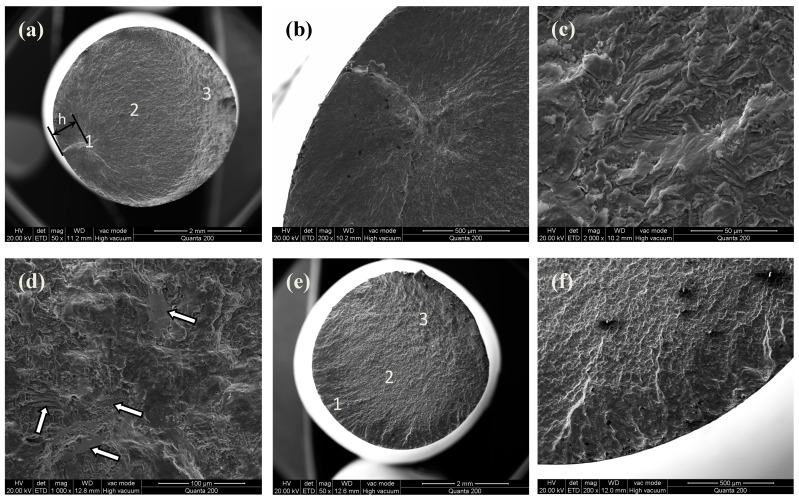
Fatigue fracture surfaces of MUSR-treated specimen at different stresses: (**a**) fracture surface at 560 MPa; (**b**) area “1” 200×; (**c**) the striations in area “1”; (**d**) the steps in area “1”; (**e**) fracture surface at 700 MPa; (**f**) cleavage striations in the surface modification layer.

The fracture morphologies of MUSR-treated and untreated samples after fatigue tests at different stress amplitudes are shown in Figure 12 and Figure 13, respectively. The fatigue cracks (Figure 12a) of untreated specimens at 450 MPa are initiated on the surface. The fracture morphology can be divided into three zones of crack propagation. The crack initiation area “1” is followed by zone “2” of stable crack propagation with apparent striations. Then, a zone “3” of accelerated crack propagation. The final fracture exhibits the failure by the nucleation, coalescence and growth of void. Generally, either slip planes of α-lamellas or the interfaces between the grains are the most frequent crack initiation sites [12]. As shown in Figure 12b, cracks in area “1” propagate crystallographically along the slip planes within the grains or across the grain boundaries appropriately oriented to the applied stress, and wide cleavages and coarse steps (indicated with the arrow) can be seen on the fracture surfaces. The fracture surfaces of Figure 12c,d are classic brittle and ductile fractures, which are composed of many micro-cracks (indicated with black arrows) and dimples. The fatigue fracture process of untreated samples at 450 MPa gradually changes from the initially brittle fracture (Figure 12c) to the final ductile fracture (Figure 12d). When the stress is increased to 700 MPa, the fracture surface of Figure 12e is similar to that of Figure 12a. The only difference is the greater number of crack initial sites and the relatively large zone “3”. Moreover, there are some wide striations at the edge of the fracture surface of Figure 12f. This should be attributed to the large applied stress accelerating the formation of the increased crack initial sites on the material surface, which results in the multiple surface cracking and rapid fracture of untreated samples. In addition the residual pores within the material also have significant influence on the crack propagation.

For MUSR-treated samples, the crack initiation sites in Figure 13a are shifted to the subsurface region due to material surface hardening layer, where is below the compressive residual stress field. There is a consistent transition layer (h distance from the surface) in Figure 9a. Then, the cracks gradually extend to the material surface and zone “2” from zone “1” (Figure 13b), and the fracture surface in the material surface layer is comparatively smooth. This illustrates that compressive residual stress, strain-hardening, grain refinement and dense material, caused by MUSR treatment, effectively prevent the generation of micro-cracks in the material surface layer. The slip planes of the α-phase are still predominant initiation sites in zone “1”. A number of tiny cleavage striations (Figure 13c) with the same orientation and several coarse steps (Figure 13d) can be observed in the fracture surface. They are formed by transgranular and intergranular fracture, respectively. Then, the cracks gradually propagate on the order of “1”–“3” (Figure 13a) until the sample fracture. When the applied stress is increased to 700 MPa, the crack initial sites are obviously increased and begin on the material surface (Figure 13e). This can be explained by the stress concentration produced by the high cyclic stress applied on the material surface. However, the crack initial sites of Figure 13e are still less than that of Figure 12e, and the striations in the material surface-modified layer (Figure 13f) are relatively small. MUSR-treated samples have similar fracture surfaces in the zones of “2” and “3” with untreated samples. The improved fatigue properties of MUSR-treated samples should be attributed to the combined effects of compressive residual stresses, stain strengthening, grain refinement and microstructure modification in the material surface layer. The fatigue limit is increased with the decreasing grain size and follows the Hall–Petch relationship in materials exhibiting planar slips. It is well known that the more randomly oriented microstructure, the lower the crack propagation rate due to crack branching and deflection, and thus the better the fatigue strength/lifetime [27]. However, crack detection is more difficult in critical applications. This will complicate the estimation of the remaining lifetime of a component.

## 4. Conclusions

(1) Surface layer mechanical properties and microstructure of Ti-6Al-4V alloy prepared by HIP are obviously improved by MUSR treatment relative to the initial state.
(2) Compared to the smaller increase extent (2.8%) of tensile strength, the fatigue strength (560 MPa) of samples after MUSR treatment has about a 25% increase on the base of 10^7^ cycles. The increased strength should be the combined effects of the increased microhardness and compressive residual stress, low surface roughness, grain refinement and micro-pore healing in the material surface layer.
(3) MUSR-treated samples show the different tensile and fatigue fracture morphologies compared to untreated samples. The tensile sample has a transition layer with the thickness of h at the edge of fracture surface, and the crack initiation sites are located in the micro pores inside the material. The fatigue sample has the crack initiation sites in the subsurface region, where is below the compressive residual stress field, and the distance of crack initial sites from the treated surface is consistent with the transition layer thickness of tensile samples.

## Figures and Tables

**Table 1:** Chemical composition of HIP (hot isostatic pressing) Ti-6Al-4V alloy (wt %).

Element	Al	V	Fe	C	O	Si	N	H	Ti
Average	6.0	4.0	0.21	0.08	0.12	≤0.05	0.09	0.002	rest

**Table 2:** MUSR (multi-pass ultrasonic surface rolling) processing parameters.

Frequency (kHz)	Load (N)	Ball Diameter (mm)	Amplitude (μm)	Spindle Speed (rpm)	Axial Feed (mm/rev)
30	600/750/900	15	10	45	0.08
